# Optimizing Nutritional Management Before and After Bariatric Surgery: A Comprehensive Guide for Sustained Weight Loss and Metabolic Health

**DOI:** 10.3390/nu17040688

**Published:** 2025-02-14

**Authors:** Evelyn Frias-Toral, Sebastián Chapela, Victoria Gonzalez, Andres Martinuzzi, Julieta Locatelli, Natalia Llobera, Ezequiel Manrique, Gerardo Sarno, Monica Mingo, Federica Marchese, Raffaele Cuomo, Ludovica Romaniello, Martina Perna, Annalisa Giordano, Biagio Santella, Luigi Schiavo

**Affiliations:** 1 Escuela de Medicina, Universidad Espíritu Santo, Samborondón 0901952, Ecuador; evelynft@gmail.com; 2 Departamento de Bioquímica Humana, Facultad de Medicina, Universidad de Buenos Aires, Ciudad Autónoma de Buenos Aires C1121ABG, Argentina; sebachapela@gmail.com; 3 Unidad de Soporte Nutricional, Hospital Británico de Buenos Aires, Ciudad Autónoma de Buenos Aires C1280AEB, Argentina; naty.llobera@gmail.com; 4 Unidad de Soporte Metabólico y Nutricional, Sanatorio Allende, Córdoba X5000BFB, Argentina; vgonzalez@sanatorioallende.com; 5 Facultad de Ciencias de la Salud, Universidad Católica de Córdoba, Córdoba X5000IYG, Argentina; 6 Unidad de Soporte Nutricional, Sanatorio Rio Negro, Rio Negro R8500BAD, Argentina; andres.martinuzzi@nutrihome.com.ar; 7 Asuntos Profesionales y Educación, Fresenius Kabi Argentina, Ciudad de Buenos Aires C1428AAU, Argentina; 8 Instituto Alexander Fleming, Ciudad Autónoma de Buenos Aires C1426ANZ, Argentina; julieta.locatelli@gmail.com; 9 Unidad de Soporte Nutricional, Hospital Privado Universitario de Córdoba, Córdoba X5016KEH, Argentina; ezequiel.manrique@hospitalprivado.com.ar; 10 Nutrihome S.A., Ciudad de Buenos Aires C1428AAI, Argentina; 11 Scuola Medica Salernitana, “San Giovanni di Dio e Ruggi D’Aragona” University Hospital, 84131 Salerno, Italy; gsarno79@yahoo.it; 12 Department of Medicine, Surgery and Dentistry “Scuola Medica Salernitana”, University of Salerno, 84081 Baronissi, Italy; mmingo@unisa.it (M.M.); federicamarchese86@libero.it (F.M.); cuomoraffaele98@gmail.com (R.C.); l.romaniello2@student.unisa.it (L.R.); m.perna6@studenti.unisa.it (M.P.); annalisa.giordano@biologo.onb.it (A.G.); bsantella@unisa.it (B.S.); 13 National Biodiversity Future Center (NBFC), 90133 Palermo, Italy

**Keywords:** abdominal obesity, central obesity, bariatric surgery, nutrition therapy

## Abstract

Obesity is associated with multiple comorbidities that contribute to increased mortality among affected individuals. There are multiple treatments for this condition, including nutritional interventions, pharmacological therapies, and surgical procedures. Within these, bariatric surgery is an effective treatment option that requires a multidisciplinary approach, both before and after surgery. Nutritional management prior to surgery aims to achieve metabolic control and reduce comorbidities associated with the procedure. Postoperative nutritional management focuses on preventing complications, ensuring adequate nourishment, and providing necessary supplementation for optimal recovery and long-term success. This narrative review examines all these critical aspects of nutritional management in bariatric surgery, including preoperative nutrition, postoperative nutrition and physical activity recommendation, different nutritional aspects according to the type of bariatric surgery, and future directions for investigation.

## 1. Introduction

Obesity is characterized by a body mass index (BMI) greater than 30 [1]. This complex pathology, with multifactorial origins, affects numerous organs and systems [2,3]. While BMI is widely used in epidemiological studies to define obesity, its sensitivity is limited. Significant inter-individual variation in body fat percentage at a given BMI is observed, explained by factors like age, sex, and ethnicity [4,5]. Obesity is associated with an increased risk of mortality and a broad spectrum of comorbidities, including type 2 diabetes mellitus, metabolic syndrome, nonalcoholic fatty liver disease, osteoarthritis, cardiovascular diseases, obstructive sleep apnea (OSA), chronic kidney disease, hyperlipidemia, hypertension, some types of cancer, and depression [1,6,7,8,9,10,11,12]. This health issue is becoming more common [1,2,4,13,14]. Since 1980, the prevalence of obesity has increased globally, with around one-third of the world’s population being classified as overweight or obese [1,15]. Although obesity was originally seen to be an issue in wealthy nations, the prevalence of children with overweight or obesity in these countries has declined or stabilized since the early 2000s [16,17]. On the other hand, overweight and obesity rates are increasing in low- and middle-income countries, particularly in urban areas [1,18,19,20,21,22]. Bariatric surgery (BS) is widely recognized as an effective intervention for reducing comorbidities associated with obesity [14,23,24,25,26,27]. For example, BS significantly improves and reduces type 2 diabetes in persons with obesity compared to traditional medical therapy [23,24,28,29]. Depending on the type of surgery, an average weight loss of 15 to 30% can be achieved, with the Roux-en-Y gastric bypass yielding the most significant results [23,24,30,31,32,33]. It was also described that BS reduces metabolic syndrome [23,24,34]. In this context, nutrition plays a critical role both preoperatively and postoperatively. In the preoperative setting, it is important to identify patients with nutritional deficiencies and implement strategies to optimize their nutritional status, thereby reducing surgical risks [35]. This review aims to comprehensively examine all the nutritional aspects of BS patients, focusing on their complex pathology and associated comorbidities, with an emphasis on preoperative and postoperative care. In this way, health personnel dedicated to patient care are offered a guide on all nutritional aspects to consider during the care of this complex pathology, as well as future considerations on new therapies.

## 2. Methodology

The current article is a narrative review. A bibliographic search was conducted prioritizing articles up to 10 years ago. Only articles found in the NIH, National Library of Medicine (PUBMED) were included. The search parameters were adjusted according to each section. Significant publications were considered for this narrative review. The search was performed via PubMed using a combination of related search terms including “Bariatric Surgery”, “Metabolic Surgery”, “Nutrition”, and “Physical Activity”. According to their pertinence, titles and abstracts of the identified articles were examined by the authors, selected for full review, and included in the actual article.

## 3. Preoperative Nutritional Management

### 3.1. Nutritional Evaluation and Screening

Evidence highlights the importance of a comprehensive nutritional evaluation prior to BS, given the high risk of malnutrition and micronutrients deficiencies among these patients [36,37,38,39]. This evaluation must be conducted by a specialist and should include detailed assessments of micronutrient levels. For malabsorptive procedures, more thorough evaluations are required compared to purely restrictive procedures [35]. A preoperatory nutritional evaluation allows for the identification and correction of nutritional deficiencies prior to surgery. This approach helps prevent postoperative complications and optimizes surgical outcomes [35]. A 2019 study found that the patients who underwent micronutrients corrections prior to surgery did not develop new deficiencies within the first year after the BS. Conversely, patients who did not address preoperative micronutrients deficiencies continued to experience deficits in one or more micronutrients after surgery [40]. Patients with obesity often present nutritional deficiencies due to several factors. Malabsorption is one contributing factor, as obesity itself may impair the absorption of certain micronutrients [37,41,42]. Frequent dieting, particularly extreme or restrictive weight-loss regimens, can also lead to nutrient deficiencies [43]. Cobalamin (Vitamin B12) deficiency is common, often linked to the use of medications for obesity-related comorbidities, such as metformin, proton pump inhibitors, angiotensin-converting enzyme inhibitors, and colchicine, with small intestinal bacterial overgrowth further exacerbating the condition [37,44]. Vitamin D deficiency is highly prevalent in adults with obesity [45,46], with a 65% prevalence rate observed in patients with obesity during the perioperative period [41,47], though supplementation has been shown to improve this deficiency [41]. Iron deficiency, affecting up to 45% of these patients in some studies, is associated with poor dietary intake, increased iron requirements, and reduced absorption in the duodenum [41,44,48,49]. A comprehensive medical evaluation is mandatory before BS to ensure patient safety and optimize outcomes. This evaluation includes a detailed medical history encompassing weight history, dietary habits, social background, psychological history (including eating disorders and substance abuse), physical activity, medication use, and psychosocial factors that may influence weight loss [35,40,50]. A thorough physical examination and complete anthropometric assessment are also essential [35,40,50,51]. Laboratory tests should evaluate electrolytes, vitamins, iron, folate, calcium levels, and a complete metabolic panel to identify potential deficiencies or abnormalities [35,40,50,51]. Additionally, a psychological assessment is critical, as psychosocial factors significantly influence long-term success following BS, including adherence to postoperative lifestyle changes, emotional adjustment, and weight loss results [35,40,50,51]. Preoperative imaging for gastrointestinal tract assessment is essential in BS candidates. An abdominal ultrasound is particularly useful for assessing biliary tract pathology, such as cholelithiasis, which is common in patients with obesity. It also helps in identifying hepatic conditions, including steatosis, fibrosis, and nonalcoholic steatohepatitis [40]. Moreover, esophagogastroduodenoscopy (EGD) provides visualization of the esophagus, stomach, and duodenum, and can detect conditions such as esophagitis, hiatal hernia, peptic ulcer, and tumors [36].

### 3.2. Preoperative Dietary Optimization

Weight loss before BS is crucial not only for improving the patient’s metabolic profile but also for enhancing the technical feasibility of the procedure [52,53]. The increased volume of the fatty liver can occupy a large part of the surgical field [52,53]. In addition, it may be prone to bleeding during manipulation to access the stomach [52,53]. Furthermore, intra-abdominal fat located at the esophagogastric junction, greater omentum, and gastrosplenic ligament, can complicate surgical techniques [52].

**Figure 1:**
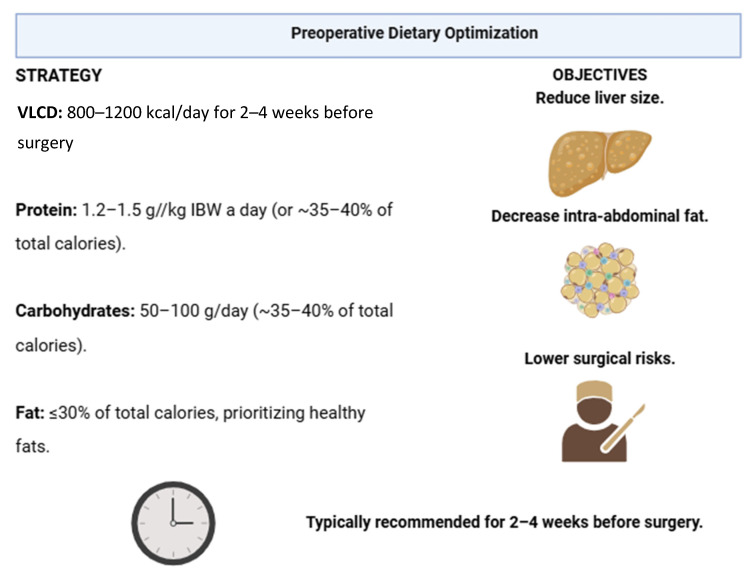
Preoperative dietary optimization: strategies and objectives for enhanced surgical outcomes. The objectives of preoperative nutrition are to reduce liver size, reduce intra-abdominal fat and decrease surgical risks [52,62,63]. VLCD: Very low-calorie diet, IBW: Ideal body weight.

Low-calorie (LCD) or very low-calorie diets (VLCD) are commonly recommended for liver size reduction and enhance operative safety in BS [54]. Many preoperative protocols include a low-calorie liquid diet for two to three weeks before surgery [50,55]. For instance, in Canada, these diets typically provide about 900 Kcal/day, consisting of low carbohydrate, high protein, and low-fat intake. BS patients are often instructed to consume commercially available protein shakes, which provide 650 to 900 kcal/day during this period. Evidence suggests that a low-calorie, low-carbohydrate diet can reduce liver volume by up to 19% and visceral adipose tissue by 17% [56]. Patients adhering to a four-week preoperative ketogenic diet supplemented with micronutrients can achieve significant reductions in body weight, left hepatic lobe volume, and micronutrient deficiencies [57]. A systematic review confirmed that VLCDs are effective in reducing body weight and liver volume before surgery. However, while VLCDs contribute to improving surgeon-perceived ease of operation, they do not significantly reduce intraoperative or postoperative risks [58]. Preoperative weight loss with a two-week VLCD can result in a weight reduction of approximately 6 kg [58]. In one study, patients who followed a VLCD providing a commercial liquid formula with 800 kcal/day for two weeks before surgery showed no significant difference in operative time compared to patients who consume the same caloric intake through a standard diet [59]. However, surgeons reported higher difficulty levels in control patients (normal food) than in VLCD patients (commercial liquid formula), and the 30-day complication rate (including deep gastrointestinal bleeding, infection, dehiscence, and anastomotic leak) was higher in the control group [59]. Another study found that patients achieving ≥8% excess weight loss through a four-week VLCD had shorter hospital stays and greater postoperative weight loss at three months and one year compared to those who did not follow a VLCD [36]. However, the degree of preoperative weight loss did not affect major complication rates or surgical conversion rates [55]. It is worth mentioning that the Mediterranean diet may also be an effective option for preoperative management. A study involving 37 male patients with obesity found that a Mediterranean-enriched protein diet reduced weight, liver size, and visceral fat but did not affect fat-free mass [60]. Although there is no consensus on the optimal preoperative diet for BS, diets exceeding three months are discouraged to maintain motivation and compliance [61]. For patients with super-obesity (BMI > 50 kg/m^2^), reducing visceral fat and liver size can facilitate the procedure and decrease surgical risks [40,55]. As a conclusion, low-calorie diets effectively reduce liver size and visceral fat, helping to prepare patients for BS. While they may enhance surgical feasibility, evidence regarding their direct impact on operative safety remains mixed [40,55]. Protein, carbohydrates, fats, and micronutrients play crucial roles in preoperative nutritional optimization. Proteins play a critical role in supporting muscle mass, wound healing, and immune function, with a recommended intake of 1.2–1.5 gr/kg of ideal body weight (IBW) per day. High-quality, easy to digest protein sources should be prioritized, and commercial liquid proteins supplements can be a convenient way to meet these requirements [36,40,55]. Carbohydrates provide energy and maintain glycogen stores, but excessive intake, particularly from simple sugars, can exacerbate fatty liver and increase surgical risk. A low-to-moderate carbohydrate intake, comprising 40–50% of total caloric intake, is recommended, with a focus on complex carbohydrates such as whole grains and vegetables [36]. Preoperative VLCDs or LCD providing 800–1200 Kcal/day are commonly used to reduce liver size and improve intraoperative conditions [36]. Fats, essential for the absorption of fat-soluble vitamins, energy production, and cellular integrity, should constitute 20–30% of total caloric intake, with an emphasis on healthy unsaturated fats from fish oil, olive oil, avocados, nuts, seeds [36]. Trans fats and saturated fats should be minimized to reduce health risks [36]. Adequate intake of essential micronutrients, including iron, calcium, vitamin D, and B vitamins, is mandatory to prevent deficiencies [36,44]. Figure 1 provides a summary of dietary optimization strategies.

### 3.3. Managing Comorbid Conditions

Patients undergoing BS often present with multiple comorbidities that must be carefully addressed to improve postoperative outcomes [64]. For patients with diabetes, achieving tight glycemic control during the perioperative period is crucial to minimize adverse events. It is recommended to maintain serum glucose levels below 150 mg/dL or HbA1C < 7% [61,65]. In patients with hypertension, continuing usual antihypertensive medications is mandatory, except for insulin, diuretics, and oral hypoglycemic agents, which should be managed on a case-by-case basis up to the time of surgery [61]. OSA is a common comorbidity in patients with obesity [66] and significantly increases the risk of postoperative complications [67]. Screening for OSA is recommended for all candidates for BS [35,50]. Common screening tools include the STOP-Bang questionnaire, which, while validated for the general population, may not effectively detect moderate-to-high-risk OSA in bariatric patients, as suggested by a 2018 study. Similarly, the Berlin questionnaire has limited reliability in identifying moderate-to-high-risk OSA in this population. The Epworth Sleepiness Scale evaluates daytime sleepiness, a common symptom of OSA, but it may not significantly correlate with the severity of the condition. Given these limitations, clinicians are encouraged to maintain a high index of suspicion for OSA in bariatric patients and to adopt a low threshold for conducting formal polysomnography (PSG), which remains the gold standard for diagnosing OSA [40]. Gastrointestinal pathologies are a critical consideration in bariatric patients, with preoperative EGD playing a debated yet significant role due to the high prevalence of gastrointestinal (GI) disorders in this population [40]. While the correlation between GI symptoms and endoscopic findings is often poor, identifying and treating underlying GI pathology before surgery is crucial [68]. EGD also facilitates the detection of Helicobacter pylori, a bacterium associated with peptic ulcers and gastric cancer development [40]. Additionally, gallbladder disease, particularly cholelithiasis, is common in patients with obesity. Preoperative abdominal ultrasound can help assess biliary tract pathology and guide appropriate management [36]. Patients with obesity, particularly those preparing for BS, have a very high prevalence of psychological disorders [69]. It is estimated around 50% of them experience depression, anxiety, and binge eating, conditions that worsen with increasing BMI and significantly impair quality of life [70]. A thorough preoperative assessment and psychological evaluation identify issues that may affect surgical success. Postoperative support is equally critical in addressing challenges such as emotional dysregulation and body image concerns [71]. Psychological factors, particularly impulsivity and mood disorders, play an important role in influencing weight loss success and the risk of weight regain [69,70]. Mental health assessment and management are recommended both before and after BS to address these conditions and support long-term outcomes [40]. Managing comorbid conditions in bariatric patients requires a multidisciplinary approach involving [72] surgeons, endocrinologists, nutritionists, psychologists, and other healthcare professionals. Effective communication and coordinated care among team members are essential to optimize patient outcomes [50].

### 3.4. Pre-Surgical Exercise Recommendations

Preparation for BS involves a comprehensive approach that includes lifestyle modifications, particularly in physical activity and nutrition, to optimize surgical outcomes and support recovery [73,74,75]. Exercise plays a crucial role in preparing candidates both physically and metabolically, improving their overall health status before undergoing surgery [73,76]. The following sections outline exercise recommendations and preparation strategies for BS candidates [73,74,77]. Recommended exercises: Both moderate-intensity continuous training (MICT) and high-intensity interval training (HIIT) are beneficial. MICT is particularly beneficial for reducing fat mass and increasing muscle mass, while HIIT can enhance adiponectin levels, contributing to improved metabolic health [73,77]. Program duration: A typical pre-surgical exercise program may consist of 10 sessions over 4 weeks, with each session lasting about 30 min, performed 2–3 times per week [73,77]. Physical and metabolic benefits: Regular exercise before surgery improves aerobic capacity, insulin sensitivity, and lipid profiles, which are crucial for reducing surgical risks and enhancing recovery outcomes [73,77]. Table 1 summarizes all preoperative management recommendations.

## 4. Postoperative Nutritional Management

### 4.1. Immediate Postoperative Phase

The main objectives of the immediate postoperative period are ensuring hydration, adequate nutrient intake (mainly protein), minimizing muscle mass loss, promoting healing, and assisting in the correct dietary progression [80]. Food reintroduction begins during hospitalization, within the first 24–48 h. If no gastrointestinal symptoms such as nausea or vomiting are present, clear liquids are introduced in small sips, increasing the volume gradually to a target of 2 L/day [81,82]. Due to the reduction in the gastric volume and the post-surgical edema, solid food intake is challenging during the first days [59]. Gradual progression in food consistency during the first week aims to minimize nausea or vomiting, which could compromise surgical integrity and patient safety [83]. If clear liquids are well tolerated, the patient is discharged with a low-fat, full liquid diet supplemented with 15–30 g of protein at lunch and dinner, along with vitamin and mineral supplementation. This diet is maintained for 7 to 14 days, depending on patient evolution and tolerance [80,84]. The low-fat liquid diet should not include sugary, fatty, caffeinated, or carbonated beverages. Sherbet intake should be limited [74,85], and drinks in large quantities or consumed with straws should be avoided to reduce air intake [85]. Diet progression 1 to 2 weeks after surgery involves transitioning to a high-protein, low-fat, and moderate-carbohydrate diet with puréed foods that do not require chewing. Protein intake remains consistent at lunch and dinner, and lactose and simple sugars are avoided. Meals are fractionated, and the duration of this phase averages 2–3 weeks, depending on patient evolution and symptoms [80,84]. Fluid intake should continue at 2 L/day, with beverages consumed 15–30 min before or after meals, avoiding sugary, carbonated, or caffeinated options [82] (Table 2).

### 4.2. Long-Term Nutritional Strategies

After the first 2 weeks, soft foods are gradually reintroduced. These should be cut into small pieces, reinforcing correct mastication. Patients transition to a fractionated, hypocaloric-hyperproteic diet with reduced portions, including all food groups [59,80]. During the initial postoperative months, portion sizes should not exceed 200 cc per meal, distributed across 4–6 meals per day [80,82]. Solid and liquid foods should not be consumed simultaneously, a guideline typically maintained for approximately four weeks [84].

According to the Recommended Dietary Allowances (RDA), a minimum intake of 130 g/day of carbohydrates is required for sufficient glucose supply to the brain and nervous system [58]. Carbohydrates should be limited to 35–50% of daily caloric intake [74], with a preference for low-glycemic-load foods [80]. The American Society for Metabolic and Bariatric Surgery (ASMBS) guidelines suggest that 10–35% of the daily caloric intake should come from protein [84]. Fat intake should align with general population guidelines, constituting 20–35% of daily calories, with a greater proportion of unsaturated fats [82,84]. Different authors suggest a slightly higher range of 35–42% [74] Caloric intake may be limited to under 1000 calories/day during the first year of postoperative surgery [74]. Patients should receive ongoing guidance and follow-up from a registered dietitian specialized in BS. This ensures appropriate dietary modifications, maximization of surgical outcomes, and minimization of complications [59,83]. Dietary advice should be addressed with regard to the implementation of new eating habits and evaluation of the amount of macronutrients ingested to avoid nutritional deficits [59,82]. Micronutrient control in the postoperative period will depend on the preoperative nutritional status, type of bariatric procedure performed, compliance with dietary recommendations, oral tolerance, and clinical symptoms. Care should be taken to avoid excessive supplementation [84]. The reduced gastric capacity following BS leads to limited food intake, intolerance to certain foods, decreased gastric acid secretion, lack of intrinsic factor, and reduced intestinal absorption surface, all contributing to micronutrient deficiencies [80]. Daily vitamin and mineral supplementation should be prescribed to each patient after the BS procedure, according to the type of surgery and current guidelines [59]. Thiamine deficiency may be due to a small thiamine reserve, rapid weight loss, poor nutritional intake, persistent vomiting, small bacterial overgrowth, or noncompliance with the indicated vitamin supplementation [81]. Wernicke’s encephalopathy is the main complication of thiamine deficiency, which typically develops within weeks or months after surgery [86,87,88]. Thiamine repletion varies according to the administration route: orally at 100 mg 2–3 times/day, intravenously at 200 mg 3 times/day (followed by 500 mg 1–2 times/day for 3–5 days), and then 250 mg/day until symptom resolution [84]. Viera de Sousa et al. [89] found considerable vitamin B12 deficiency in those patients undergoing RYGB, 17.46% and 16.74% at 6 and 12 months post-surgery, respectively. However, the study showed difficulties in accurately assessing compliance with postoperative supplementation. Vitamin B12 absorption is affected by the following: (1) Reduced gastric acid and pepsin affecting the digestion of cobalamin-binding proteins; (2) limited release of vitamin B12 from its complex with protein R due to shortened contact time with pancreatic proteases; (3) inadequate uptake by intrinsic factor produced by gastric parietal cells; (4) food intolerance to vitamin B12-rich sources like meat; and (5) small bacterial overgrowth [89]. It is a common cause of anemia and neurological symptoms [86]. Vitamin B12 repletion is recommended at 1000 μg/day until normal laboratory values are achieved [84]. Annual monitoring is crucial, especially in patients on medications that exacerbate deficiency risks, nitrous oxide, neomycin, metformin, colchicine, proton-pump inhibitors, and seizure medications [84]. Iron deficiency is caused by decreased intake, high prevalence of intolerance to iron-rich foods, reduced gastric acidity, and surgical bypass of the duodenum, the primary site of iron absorption [86,90]. Its deficiency produces microcytic and hypochromic erythrocytes with reduced oxygen-carrying capacity [86]. It also causes symptoms such as fatigue, hair loss, headaches, restless legs syndrome, cognitive, and attention deficits even in the absence of anemia [90]. Iron deficiency anemia is more common after RYGB than sleeve gastrectomy (SG) [89]. Their status should be monitored in all patients within 3 months after BS, every 3–6 months during the first year after, and annually thereafter. Repletion could be treated with oral doses of 150–200 mg/day of elemental iron, or up to 300 mg 2–3 times/day as needed [84]. Vitamin D deficiency is the most prevalent micronutrient deficiency pre- and post-surgery, with rates of 74.24% in SG patients and 62.05% in RYGB patients [89]. An initial and annual post-surgical evaluation of vitamin D is recommended after RYGB, SG, or BPD/DS [84]. Calcium absorption is impaired due to reduced meal mixing with bile and pancreatic enzymes, often exacerbated by vitamin D deficiency. Hypocalcemia can result in secondary hyperparathyroidism, leading to bone loss and increased fracture risk [84]. Both deficits, calcium and vitamin D, are related due to the role of the latter in calcium transport [89]. Recommended supplementation includes 3000–6000 IU/day of vitamin D3 and 1200–1500 mg/day of calcium for SG and RYGB, increasing to 2400 mg/day for biliopancreatic diversion with duodenal switch (BPD/DS) procedures [84]. Furthermore, BS can impair the absorption of fat-soluble vitamins (A, D, E, K) due to disrupted bile acid and pancreatic enzyme activity [91]. To address potential deficiencies, it is recommended to administer 10,000–25,000 IU/day of vitamin A for patients with deficiencies but without corneal changes. For patients with corneal changes, doses of 50,000–100,000 IU/day intravenously for three days, followed by 50,000 IU/day for two weeks, are suggested. While the therapeutic dose for vitamin E is not yet clearly defined, supplementation of 100–400 IU/day is generally recommended. For vitamin K, an intravenous dose of 10 mg is advised in cases of acute malabsorption, while in cases of chronic malabsorption, 1–2 mg/day orally or 1–2 mg intravenously per week is recommended [84]. Lifelong biochemical monitoring and supplementation are essential for BS patients [81]. The format of the supplement could be chewable, powder, or liquid for the first 3 months, then capsules according to tolerance [85]. Evaluating drug–nutrient interactions is important to prevent micronutrient deficiencies with drugs such as antacids, fluoroquinolones, histamine H2 antagonists, laxatives, loop diuretics, metformin, metoclopramide, proton pump inhibitors, thiazides, and tetracyclines [92].

### 4.3. Protein Intake

Eating sufficient quantities of protein is thought to prevent lean body mass loss during rapid weight loss [59,62]. Adequate protein intake also prevents hair loss and peripheral edema [93]. Protein malabsorption is a common macronutrient deficit post-surgery, as a result of anatomical alteration, decreased intake, impaired digestion and absorption [91]. In addition, inadequate intake (≤60 g/day) may reduce satiety and diet-induced thermogenesis, potentially hindering weight loss, although the evidence for a high-protein diet in preserving lean mass was scant, largely due to the low compliance rates with protein intake observed in most of the reviewed studies [94]. ASMBS guidelines recommend a daily consumption of at least 60 to 120 g with higher intakes of up to 2.1 g/kg of IBW for certain individuals [84]. Busetto et al. [83], suggest a minimum intake of 60 g of protein/day and above 1.5 g/kg/day of IBW. Other authors recommend a daily protein intake of 60 to 80 g or 1 to 1.5 g/kg of IBW [81,95]. Dagan et al. [82] recommend increasing to 90–120 g/kg/day of IBW after BPD or BPD/DS. Protein intake is frequently and substantially reduced, mainly in the first-year post-surgery, due to food intolerances and preferences for non-protein foods [59,83]. For this reason, protein supplements (30 g/day) are suggested to ensure adequate intake [83]. Follow-up by a qualified registered dietitian is essential to monitor and adjust protein intake during the postoperative phases [85]. To achieve protein intake recommendations, foods rich in protein should be placed over those rich in carbohydrates or fats [62,82]. Suggested protein sources include low-fat dairy products, egg whites, lean meats, soy products, lentils, and hard cheeses. Whey protein supplements are also effective for increasing leucine intake, which supports muscle synthesis [80,82].

### 4.4. Preventing Common Postoperative Complications

Postoperative nausea and vomiting may result from rapid ingestion, inadequate chewing, mixing of solids with liquids, or consuming fibrous foods that can produce obstruction [80]. The formation of phytobezoars, linked to reduced gastric motility, pyloric dysfunction, and hypoacidity, necessitates caution with high-insoluble-fiber foods. Hydration and thorough chewing are essential [82]. In the presence of diarrhea, fiber should be excluded from the diet for 3 days, ensuring proper hydration, and gradually reintroduce fiber if constipation occurs, focusing on cooked, easily digestible sources [80]. The modification of the gastric anatomy and innervation, due to the intervention, may allow undigested food to reach the small intestine too quickly, producing the so-called Dumping syndrome [96]. There are two types of Dumping syndromes. The early Dumping syndrome occurs within 15 to 30 min post-ingestion and is characterized by gastrointestinal symptoms such as abdominal pain, nausea, diarrhea along with vasomotor symptoms including fatigue, desire to lie down after ingestion, palpitations, sweating, tachycardia, hypotension, and rarely, syncope [80]. These symptoms result from the arrival of hyperosmolar contents into the small intestine, causing a shift in fluid from the intravascular compartment into the intestinal lumen, resulting in the release of several gastrointestinal peptide hormones, duodenal or jejunal distension, and a reduction in circulating blood volume [96]. The late Dumping syndrome occurs 1 to 3 h after food intake, presenting as hypoglycemia when blood glucose levels reach their nadir [59]. This condition is likely related to changes in the secretion of gastrointestinal hormones and insulin [83], with hyperinsulinism produced by incretin release after carbohydrate intake [96]. The management of this syndrome involves dietary modifications, including eliminating simple sugars and replacing them with complex carbohydrates, increasing fiber and protein intake, fractionating meals, reducing portion sizes, and avoiding fluid intake during meals by delaying fluids until 30 min after eating solid foods [80,81]. Hypoglycemia can also be prevented by drinking half a glass of orange juice (or an equivalent sugar source) approximately one hour after ingestion [83]. Common after RYGB postprandial hypoglycemia results from hyperinsulinemia driven by glucagon-like peptide 1 (GLP-1) [59] and reduced insulin clearance [97]. Oral carbohydrate intake (10 to 15 g) could be recommended to alleviate symptoms [98]. To prevent hypoglycemia events, one study found that distributing carbohydrates across six meals, with an intake of 30 g per meal, significantly decreased the number of hypoglycemia events in patients who underwent RYGB [99]. However, 34% of patients required additional treatment despite following a carbohydrate-restricted diet, raising doubts about the practical application of the dietary recommendations. While this highlights limitations in its application, the study demonstrated the efficacy of a structured carbohydrate-restricted diet as the primary treatment option for postprandial hypoglycemia in RYGB patients. Furthermore, the quantitative data convincingly showed a significant reduction in hypoglycemic episodes with this dietary approach [99]. Additionally, the presence of hypoalbuminemia highlights the need for an individualized assessment of dietary protein intake by a specialized dietitian [100].

### 4.5. Long-Term Follow-Up and Monitoring

Dietary deficits can result from typical food intolerances. Depending on the specific intolerance, modifications in food selection and consistency may be necessary [80]. Commonly problematic foods include meats, bread, dairy products, fibrous vegetables, rice, and pasta [83]. Adapting eating habits to the new gastrointestinal anatomy and physiology requires guidance from an expert dietitian who can offer suitable alternatives [83]. Protein malnutrition is a serious complication that can develop due to changes in eating habits secondary to intolerance to protein-rich foods [100]. It has been proposed that a total alimentary limb length of more than 300 cm in Roux-en-Y gastric bypass (RYGB) patients is necessary to prevent severe hypoalbuminemia [100]. Following BS, hunger is typically reduced, postprandial fullness increases, and changes in taste and smell can alter food preferences, resulting in portion reduction and decreased macronutrient and micronutrient intake. The prevalence of appetite changes has been observed to decrease over time after SG, whereas no temporal effect has been noted for RYGB. However, the study did not explore long-term effects beyond the 90-day postoperative period, which could provide additional insights into the sustainability of these changes [101]. Enhanced sensitivity to sweet tastes has been observed, potentially linked to GLP-1 release following sweet food consumption. A study in mice showed that taste bud cells release GLP-1 in response to sweet compounds, indicating its role as a neurotransmitter in taste signaling [102]. Regular monitoring aids in the identification of slowed weight loss, plateaus, or weight regain, facilitating timely interventions through lifestyle modifications [103,104]. The prevention, detection, and treatment of vitamin and mineral deficiencies is one of the key pillars in long-term postoperative care [59]. Daily multivitamin and mineral supplementation is recommended to avoid micronutrient deficiencies [91]. Periodic laboratory controls are suggested to identify deficiencies and enable individualized supplementation [59]. Initial monitoring should occur monthly for the first few postoperative months, then every 3 months until the end of the first year [80]. Failure to attend postoperative follow-ups during the first year has been associated with an increased risk of severe nutritional deficits, which in turn correlates with a significantly higher mortality rate due to severe nutritional complications. The study cohort primarily consisted of Caucasian women undergoing RYGB, which may limit the generalizability of the findings, especially to SG patients [105]. The nutritional management of patients undergoing BS requires the intervention of an experienced bariatric dietitian [83] to promote lasting dietary changes and optimize surgical outcomes [91].

## 5. Nutritional Challenges and Considerations for Specific Bariatric Procedures

**Figure 2:**
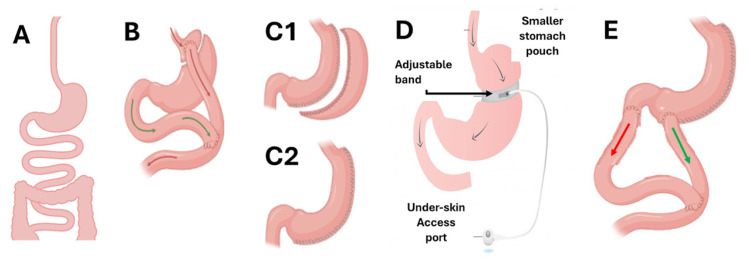
Schematic types of BS procedures. (**A**). Normal anatomy. (**B**). Roux-en-Y Gastric Bypass. (**C1**). Sleeve gastrectomy with excised stomach. (**C2**). New stomach after Sleeve gastrectomy. (**D**). Adjustable gastric band. (**E**). Biliopancreatic diversion and duodenal switch [23,24,52,63]. Red arrow: Alimentary limb. Green Arrow: Bile limb.

There are four main types of BS procedures (Figure 2). The most commonly used techniques are laparoscopic Roux-en-Y gastric bypass (LRYGBP) and laparoscopic sleeve gastrectomy (LSG) [33,106,107]. Adjustable gastric banding (AGB) is also an option, although it has been gradually replaced by sleeve gastrectomy [106,107]. The biliopancreatic diversion with duodenal switch (BPD/DS) is the least frequently performed procedure. The choice of surgical technique depends on the medical team’s assessment and the individual characteristics of the patient [79,106,107,108]. While all of these techniques are effective for weight reduction and metabolic control, each presents specific nutritional challenges and considerations [109].

### 5.1. Roux-en-Y Gastric Bypass (RYGB)

Short-term complications of BS include food intolerance syndrome, micronutrient deficiencies, dietary changes, and hypoglycemia, all of which increase the risk of malnutrition [110]. Food intolerance syndrome, often accompanied by a vitamin B1 deficiency, is characterized by frequent vomiting, typically three or more episodes per day [110]. The symptoms and severity of the condition are directly related to the extent of electrolyte imbalance and dehydration caused by vomiting and inadequate food intake [110]. In more severe cases, this condition can progress to aphagia, hypovolemia, and acute renal failure [110]. Additionally, vitamin B1 deficiency, resulting from decreased absorption and insufficient intake, can lead to neurological and cardiovascular symptoms. Wernicke’s encephalopathy, a serious complication in this population, occurs at a rate of up to 4.2 cases per 100,000 RYGB procedures [111]. It is typically characterized by ataxia, ophthalmoplegia, and altered levels of consciousness, and can be fatal in some instances [111]. Micronutrient deficiencies are particularly pronounced after RYGB, as the bypassed proximal intestine is the primary site of absorption for copper, vitamin D, calcium, and iron [112]. This is why supplementation should begin immediately after surgery and be continued for life, with proper follow-up and ongoing monitoring to detect potential deficiencies [110,113]. Hypoglycemia and eating disorders are additional problems that may arise in these patients due to anatomical changes induced by surgery [114,115]. Following RYGB, food passes more rapidly into the intestine, causing a sharp rise in glucose levels after ingestion, followed by higher peaks and lower basal levels [114,115]. This process results in increased postprandial production of GLP-1 and insulin [114,115]. The metabolic changes enhance peripheral glucose uptake and utilization while decreasing insulin clearance [114,115]. Additionally, in patients predisposed to hypoglycemia, alterations in hepatic glucose metabolism have been observed, including decreased portal glucose uptake due to disrupted signaling and glucose gradients, as well as a reduction in hepatic glucose release into the bloodstream [114,115]. The incidence of these disorders is difficult to establish, as there is no consensus on cutoff values for their definition or the clinical significance of asymptomatic hypoglycemia [114]. Postprandial hypoglycemia is not adequately defined, with physiological responses observed with blood glucose levels as low as 60 mg/dL. In contrast, fasting hypoglycemia is defined as a glucose level below 50 mg/dL [114]. The Whipple triad is used for diagnosis and includes hypoglycemia (capillary glucose less than 50 mg/dL and plasma glucose less than 55 mg/dL), compatible symptoms, and symptom resolution following correction of the metabolic disorder [114]. Management of postprandial hypoglycemia focuses on dietary modifications, such as carbohydrate restriction to avoid postprandial glucose spikes and ensuring adequate protein and fat intake [114]. Medications like sucrose can be used, and in more severe cases, somatostatin analogs have shown good results. In cases where the condition persists despite dietary and pharmacological interventions, surgical treatment, including bypass reversal, should be considered [114]. Dietary changes resulting from BS may lead to greater-than-expected weight loss, with a risk of malnutrition, which is multifactorial [116,117]. Rapid weight loss can cause alterations in body image perception, potentially resulting in disorders such as anorexia. Hormonal and gastrointestinal secretion changes affect appetite and satiety, decreasing food intake, regardless of the surgery’s mechanical effects [116,117]. Furthermore, alterations in the intestinal microbiota, combined with these factors, have been shown to influence taste and food preferences [116,117,118]. To prevent these issues, continuous follow-up by a multidisciplinary team is essential to provide adequate support and education [119,120,121]. Another complication, particularly in patients who have undergone RYGB, is exocrine pancreatic insufficiency, with an incidence ranging from 9 to 10% to as high as 40%, depending on the study and diagnostic criteria [112,122]. Clinical manifestations vary from mild symptoms, like colic and diarrhea, to severe intestinal failure with malnutrition, requiring parenteral nutritional support [112,122]. Diagnosis should be based on clinical suspicion in patients with steatorrhea and potential malabsorption. Measuring fecal elastase-1 (FE1) levels is helpful, as values below 200 micrograms/gram of stool are suggestive of the condition, though higher values do not exclude it [112,122]. The 13C mixed triglyceride breath test offers an alternative diagnostic option but is not often available. Confirmation can also be achieved through steatocrit measurement and the patient’s response to pancreatic enzyme therapy [112,122], with clinical improvement nearly confirming the diagnosis [112,122].

### 5.2. Sleeve Gastrectomy and Adjustable Gastric Banding

LSG and AGB are procedures with similar nutritional profiles, with LSG demonstrating greater effectiveness [123]. In general, they present the same nutritional challenges as RYGB, but with less frequency, probably due to the less extensive anatomical alterations involved [124]. The most characteristic micronutrient deficiencies associated with these procedures include vitamins B1, B2, D, and E, folic acid, and iron [124]. While supplementation is mandatory for patients undergoing these procedures, recommendations differ from those for RYGB patients. For LSG and AGB, supplementation is recommended for the first two years, after which individualized assessment and management are required due to the lack of clear long-term guidelines [123,125]. Mechanical restriction, combined with neuroendocrine changes, increases the risk of excessive weight loss and greater-than-expected lean mass loss in these patients, potentially leading to malnutrition and sarcopenia [126].

### 5.3. Biliopancreatic Diversion with Duodenal Switch (BPD/DS)

BPD-DS is characterized by a significant alteration in fat absorption due to delayed mixing of bile and pancreatic enzymes. This reduces enzymatic activation, thereby decreasing caloric intake and facilitating desired weight loss [127,128]. However, there is also an alteration in the absorption of fat-soluble vitamins [128,129]. Vitamin D deficiency can lead to phosphocalcic metabolism disorders and osteoporosis, while impaired absorption of Vitamin E may cause hepatic metabolic disorders, although severe consequences are rare and primarily documented in case reports [128,129,130]. Deficiencies in B-complex vitamins, iron, and folic acid are also common, manifesting as anemia and/or neurological symptoms [129]. Additionally, depending on the degree of malabsorption, many patients suffer from exocrine pancreatic insufficiency, sarcopenia, and severe malnutrition, often requiring rehospitalizations and intravenous supplementation [129,130,131]. Management of these patients requires lifelong supplementation with trace elements and vitamins, as well as multidisciplinary follow-up to ensure proper monitoring, education, and intervention. In severe cases, surgical treatments, such as bypass reversal or conversion to RYGB may be required [129,130,131].

## 6. Role of Nutrition in Long-Term Weight Maintenance and Metabolic Health

### 6.1. Importance of Adherence to Nutritional Recommendations

Contemporary nutritional strategies published by the European Association for the Study of Obesity (EASO), the Association for the Study of Obesity on the Island of Ireland (ASOI), and the Brazilian Association for the Study of Obesity and Metabolic Syndrome (ABESO) as well as the Preventing Overweight Using Novel Dietary Strategies (POUNDS Lost) study emphasize personalized interventions, regulation of energy deficits, and the promotion of balanced diets that include high-quality foods such as fruits, vegetables, and lean proteins [132]. Integration of nutritional strategies with behavioral interventions, such as cognitive-behavioral therapy, enhances adherence and promotes lasting results in weight management. Standardized recommendations for weight reduction techniques and non-weight-related outcomes are currently lacking. Nonetheless, implementing reliable frameworks for evaluating therapy effectiveness could improve treatment results [132]. Micronutrients play a crucial role, as common deficiencies in vitamins B12, D, and iron can adversely affect overall health [133]. It has been observed that adherence to supplementation decreases over time, particularly among younger patients or those with mental health disorders. It should be noted that assessing adherence using pharmacy refill data has limitations, including the lack of daily dispensing details and potential delays in detecting treatment discontinuation [133,134]. This underscores the importance of continuous medical monitoring to prevent complications, such as anemia or severe vitamin deficiencies [135,136]. Additionally, adherence to healthy habits, such as separating between liquids and solids during meals and favoring protein-rich foods, often declines over time, complicating long-term success. The strengths of this study involve reliable instruments and multiple assessment points post-surgery. However, limitations include data collected during the COVID-19 pandemic, lack of objective measurements for nutrient intake, and potential reporting bias [134]. Factors such as the cost and accessibility of care also pose barriers to adherence [136,137]. In summary, the success of weight management strategies after BS relies on a multidisciplinary approach that includes tailored nutritional interventions, continuous medical monitoring, and psychological support. Preoperative education and the promotion of sustainable dietary and lifestyle habits are essential for preventing complications, including weight regain, and for optimizing long-term outcomes [132,136]. However, standardized frameworks for evaluating various weight loss techniques and their non-weight-related effects are currently lacking.

### 6.2. Role of Physical Activity

A key component for optimizing outcomes following BS is physical exercise, which helps with energy expenditure, lean mass preservation, and weight regain prevention [125,138]. The study by Sundgot-Borgen et al. has several limitations, including reliance on specific clinical follow-ups for nadir weight, the potential impact of the COVID-19 pandemic on sedentary behavior, and underpowered analyses that may have underestimated group differences and weight recurrence associations [139,140]. Low levels of physical activity and prolonged sedentary behavior are associated with poorer metabolic and cardiovascular outcomes, as well as increased mortality risk, as demonstrated in studies by Romagna et al. and Kaouk et al. Nevertheless, significant limitations in both studies, such as inconsistent definitions, cross-sectional design, self-reported data, outdated surgical procedures, and lack of investigation into weight recovery-influencing factors, hinder the reliability and comparison of their findings [141,142]. Regular exercise dramatically improves body composition by lowering bone mass and body fat loss while improving muscle strength, VO2 max, and fat oxidation. The study by Bellicha et al. has notable strengths but also limitations, including a single reviewer and heterogeneous interventions. Among the limited studies comparing aerobic and combined training, preliminary evidence suggests that combined training is more effective. However, further research is needed to substantiate these findings [74,143]. It has been demonstrated that protocols that incorporate both resistance and aerobic training are especially successful, particularly for 16 weeks or longer regimens [74]. Despite these advantages, a large number of BS patients fail to meet the recommended 150 min of weekly physical activity, underscoring the need for strategies to improve adherence [140]. Exercise also positively impacts behavioral and psychological factors. Self-efficacy, both coping and recovery, plays a significant role in planning and adhering to physical activity regimens. Interventions aimed at enhancing these psychological factors are essential for fostering long-term self-management of physical activity. However, Maghsoodlo et al.’s study has several limitations, including reliance on self-reported diet and activity, exclusion of social factors in the Health Action Process Approach (HAPA) model, and a biased sample, limiting generalizability [144]. Conversely, high motivation levels, often driven by weight loss goals, improve adherence to both dietary and exercise recommendations, leading to better metabolic outcomes [145]. In post-bariatric patients, protein supplementation and resistance training are particularly effective ways to maintain muscle mass, increase strength, and preserve physical function while reversing lean mass loss linked to gastric bypass. Nevertheless, this study also has many shortcomings, including a substantial loss to follow-up, no random assignment to the resistance training groups, and no assessment of baseline physical activity levels [146,147]. Proper hydration is critical due to the increased risk of dehydration in this population [147]. Structured therapies, including 12-week supervised exercise programs, have demonstrated further improvements in physical function, strength, and body composition, particularly during the crucial 12- to 24-month postoperative period when weight regain is more likely [135]. To optimize the advantages following BS, consistent exercise, a healthy diet, and psychological support must be combined into a comprehensive approach. Individualized strategies should account for behavioral and sociodemographic variables that could affect adherence and results [142,144]. According to these results, encouraging active lifestyles is crucial for enhancing quality of life and preventing long-term complications in this population [132,141,148,149,150].

### 6.3. Addressing Weight Regain

**Figure 3:**
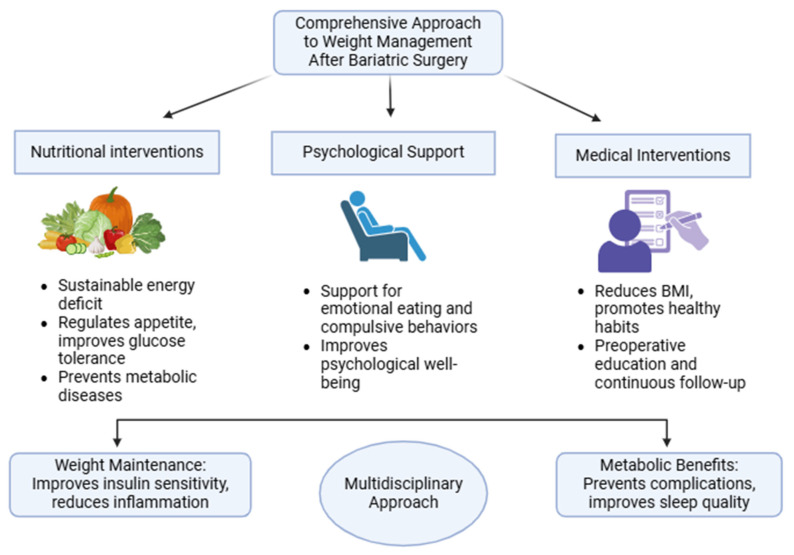
Comprehensive approach to weight management after BS [23,24,52,63]. A multidisciplinary approach is essential for the success of these interventions. This requires nutritional interventions, psychological support, and medical interventions.

Weight regain after BS is a multifactorial challenge requiring a comprehensive approach involving nutritional, psychological, and medical interventions [151,152,153]. According to the study by Lins Berber et al., major contributors include inappropriate eating behaviors, psychological disturbances, inadequate adherence to follow-up, and metabolic changes. However, the study has some limitations, such as difficulty contacting patients years after surgery, reliance on self-reported assessments, and the inability to draw causal inferences or perform robust analyses due to its design [154,155]. Behaviors like grazing, reported in up to 67% of patients, are linked to lower weight loss rates and increased weight regain [154]. Regular postoperative follow-up, including medical consultations and nutritional counseling, plays a protective role by reducing BMI and promoting healthy habits. Nevertheless, there may be a case of reverse causality, as individuals who have already lost weight might be more inclined to consult a dietitian to receive positive reinforcement for their progress [132,156]. Individualized nutritional therapy emphasizing a sustainable energy deficit and high-quality food is essential. Balanced dietary patterns, like the Mediterranean and DASH diets, support weight maintenance and provide cardiometabolic benefits [132,157,158]. These strategies, combined with low-glycemic, high-fiber diets, regulate appetite, improve glucose tolerance, reduce visceral fat, and lower metabolic disease risks [132]. Fiber intake enhances gut flora diversity, increasing short-chain fatty acid production, which reduces chronic inflammation [159,160], and improves sleep quality and mental well-being [132]. Similarly, low-glycemic, high-protein diets effectively control appetite, improve metabolic health, and increase insulin sensitivity. These metabolic benefits help counteract the reduced energy expenditure observed after BS, contributing to weight maintenance [132,140]. Psychological factors, like cognitive decline, emotional and binge eating, significantly hinder outcomes, affecting up to 65% of patients during weight regain. This underscores the need for psychological support within a multidisciplinary approach [140]. Poor dietary habits, including high fat or alcohol consumption, raise the risk of weight regain, while high-protein, low-fat diets promote weight maintenance [142]. Healthcare professionals, including dietitians and psychologists, are crucial in preventing metabolic complications and improving quality of life. Preoperative education, continuous follow-up, and sustainable dietary patterns tailored to individual needs are essential for long-term success [132,135]. In conclusion, addressing weight regain requires multidimensional strategies that combine nutritional, psychological, and medical support. These strategies optimize surgical outcomes, promote sustainable weight loss, and improve long-term patient health and well-being (Figure 3 and Table 3) [74,135].

## 7. Future Directions and Research Gaps

The most effective treatment for individuals with extreme obesity is BS, which also improves quality of life, reduces cardiovascular events, resolves or remits obesity-related comorbidities, and causes considerable and long-lasting weight loss [162]. However, a substantial proportion of patients experience suboptimal weight loss (SWL) or weight return (WR), reducing the expected benefits [163]. After BS, between 11% and 22% of patients experience SWL, defined as failing to lose 40% to 60% of the initial extra body weight within 1 to 2 years post-surgery [163]. On the other hand, WR is more prevalent in the bariatric population. However, inconsistencies in defining WR complicate the estimation of its prevalence [163]. A recently published retrospective observational cohort study found that treatment with BMS, compared to first-generation GLP-1 receptor agonists (GLP-1RA), was associated with greater mortality reduction in diabetic patients with a disease duration of 10 years or less. This benefit was mediated by more significant body weight loss [164]. In contrast, no differences were observed in the incidence of non-fatal major adverse events or mortality risk among patients with a longer duration of diabetes across treatment regimens [164]. Also, a recent meta-analysis shown that high doses of liraglutide are an effective weight reduction medication in individuals who did not respond well to BS [162]. Moreover, a retrospective study compared the efficacy of liraglutide 3.0 mg daily, a dose approved for obesity treatment, with semaglutide 1.0 mg weekly, approved for type 2 diabetes treatment, for managing recurrent weight gain after bariatric and metabolic surgery [165]. Semaglutide 1.0 mg weekly showed superior weight loss outcomes compared to liraglutide 3.0 mg daily, regardless of the type of BS procedure [165]. Despite the previously described evidence, most research on pharmacotherapy for WR consists of small open trials or retrospective studies. Additionally, individuals who have undergone bariatric surgery (BS) tend to lose less weight when taking anti-obesity medications [163]. The efficacy of cost-effective pharmacotherapy, combined with lifestyle modifications, for managing WR after BS needs to be validated through randomized controlled trials [163]. Finally, responses to BS vary widely; this may be related to different surgical techniques as well as genetic background [166,167,168]. The range of physiological reactions in the same environment is caused by genetic variance among individuals, which also explains why some people are more likely than others to gain or lose weight in the same setting following BS [166,169]. Nutritional genomics, encompassing disciplines such as nutrigenetics and nutrigenomics, is a promising field for understanding these variations [91,170]. The study of intrinsic genetic variations that explain a person’s dietary needs and forecast their likelihood of disease susceptibility is known as nutrigenetics. This includes the characterization and identification of gene variants associated with differential nutrient responses [170,171]. In 2016, Novais et al. demonstrated that the 5-HT2C gene polymorphism (rs3813929) is associated with a greater percentage of excess body weight loss following RYGB [172]. Additionally, a 2018 study found women with extreme obesity carrying the MC4R rs17782313 polymorphism tend to have higher BMIs before surgery, are less likely to achieve a non-obese BMI (<30 kg/m^2^), and typically maintain a BMI > 35 kg/m^2^, indicative of treatment failure [173]. A 2023 study further demonstrated that certain SNPs and genetic models may be helpful indicators of the body weight trajectory after BS [174]. Specifically, two SNPs in the UCP2 gene (Ala55Pro and −866G > A) have been identified as potential markers of weight reduction one year after RYGB [166,175]. In addition, Seip et al., evaluated 330 SNPs in genes involved in metabolic regulation and identified many genes that might be potential markers to distinguish changes in BMI one year after LAGB or RYGB [176]. Emerging research highlights the role of epigenetic modifications, such as DNA methylation, in predicting metabolic outcomes after BS [177,178]. For instance, specific methylation sites cg11445109, cg19469447 (annotated in the CYP2E1 gene), and cg25828445 (annotated in the NIFKP3 gene) could aid in forecasting metabolic state and serve as useful indicators of responsiveness to BS [177]. The ability to predict patient outcomes after bariatric and metabolic surgery through changes in DNA methylation is still under study [179]. Further studies are needed to determine whether pre- and post-surgical epigenome profiles can help predict outcomes in individuals with extreme obesity [179]. Also, BS may impact the expression of several genes involved in various metabolic pathways. A recent transcriptomic analysis identified approximately 1366 genes with differential expression in the postoperative phase compared to the preoperative period of RYGB [179]. These genes are linked to immunological functions, oxidative stress, substrate oxidation, adipocyte differentiation, gene transcription, lipid and energy metabolism, and cell differentiation [180,181]. To identify biomarkers for more individualized weight-loss treatments, additional research is required to assess and discover the relationships between these genetic variations and the results of BS. Vitamin deficiencies are frequent following BS, despite the routine recommendation of high-dose supplementation [182]. Finding someone with genetic variations related to vitamin and mineral metabolism may help lower the risk of micronutrient deficiencies, which can result in a number of health disorders [182]. Currently, dietitians offer individualized dietary recommendations based on phenotype and dietary patterns; genotype-based recommendations remain less common. By analyzing biological markers for vitamins and minerals and determining an individual’s “nutrigenomic profile”, it may become possible to implement more personalized micronutrient supplementation strategies for BS patients [182]. To summarize, nutritional genomics offers a promising approach for improving outcomes in patients undergoing BS. However, additional research is needed in this area, with the aim of developing highly personalized nutritional strategies and tailored recommendations to optimize care [166,170,182].

## 8. Conclusions

Patients with obesity have multiple comorbidities. BS is an option for weight loss and metabolic control. However, optimizing outcomes requires comprehensive preoperative and postoperative care. These patients prior to surgery have specific nutritional deficiencies, such as vitamin D, B12, and iron, which must be corrected. Preoperative evaluation requires staging with digestive endoscopy and abdominal ultrasound to rule out associated pathologies. After surgery, a strict diet is required, with gradual progression to minimize complications. The anatomical and physiological changes induced by surgery not only alter food intake but also contribute to specific nutritional deficiencies. Therefore, ongoing supplementation and close monitoring are essential for ensuring optimal recovery and long-term health. Personalized nutrition plays a key role in tailoring interventions to individual needs, promoting better outcomes and patient satisfaction. Achieving these goals requires a multidisciplinary healthcare team that addresses the complex needs of BS patients from multiple perspectives, both before and after surgery. Among the challenges associated with BS, weight regain is a significant concern. GLP-1 analogs offer a potential solution to mitigate this issue. Furthermore, conducting studies to compare the long-term effectiveness of metabolic surgery and GLP-1 analogs in achieving sustained weight loss and metabolic control would be highly valuable.

## Figures and Tables

**Table 1:** Comprehensive preoperative management strategies in bariatric surgery: key nutritional and clinical considerations.

	Key Points
(A) Nutritional Evaluation and Screening [35,40,50,51]	Comprehensive nutritional evaluation is critical due to the high risk of malnutrition and micronutrient deficiencies in bariatric surgery (BS) patients. Malabsorptive procedures require more thorough evaluations than restrictive ones. Preoperative corrections of deficiencies prevent postoperative complications and optimize outcomes. Obesity-related factors contribute to deficiencies, e.g., malabsorption, dieting, and medication use.
Common Deficiencies	Vitamin B12: Linked to medication use (e.g., metformin) and small intestinal bacterial overgrowth. Vitamin D: 65% prevalence during perioperative period; supplementation improves levels. Iron: Affects up to 45% of patients; caused by poor intake and reduced absorption.
Preoperative Medical Evaluation	Mandatory for ensuring safety and optimizing outcomes. Includes: Medical history: Weight, diet, psychological background, etc. Physical examination and anthropometric assessment. Laboratory tests: Electrolytes, vitamins, iron, calcium, metabolic panel. Psychological assessment: Essential for addressing long-term success factors.
Preoperative Imaging	Abdominal ultrasound: Assesses biliary tract pathology and hepatic conditions (e.g., steatosis). Esophagogastroduodenoscopy (EGD): Detects esophagitis, hernia, ulcers, and tumors.
(B) Preoperative Dietary Optimization [36,40,55]	Importance: Weight loss improves metabolic profile, reduces liver size, and enhances technical feasibility of BS. Liver size reduction (up to 19%) and visceral fat loss (17%) improve surgical conditions.
	Diets common recommendations: Low-calorie (LCD) or very low-calorie diets (VLCD) Mediterranean diet.
Micronutrient Supplementation [78]	Vitamin B12: - Oral: 1000–2000 mcg daily (SL or oral). - IM: 1000 mcg monthly (if absorption is impaired or deficiency is severe). Vitamin D: - Deficiency (serum levels <20 ng/mL): High-dose supplementation: 50,000 IU weekly for 8–12 weeks. - Insufficiency (serum levels 20–30 ng/mL): 1000–2000 IU daily, adjusted based on follow-up levels. - Maintenance dose: 800–2000 IU daily. Iron: - Oral supplementation: 45–60 mg of elemental iron daily (ferrous sulfate, gluconate, or fumarate) with vitamin C (250–500 mg), which is often co-administered to enhance absorption. - IV supplementation (if oral iron is poorly tolerated or severe deficiency is present). Dosage depends on severity; ferric carboxymaltose 1000 mg can be administered over 1–2 sessions.
Macronutrient Guidelines	Proteins: 1.2–1.5 g/kg of ideal body weight; supports muscle maintenance, healing, and immunity.
	Carbohydrates: 40–50% of intake; focus on complex carbs to avoid fatty liver.
	Fats: 20–30% of intake; prioritize healthy fats and minimize trans and saturated fats.
	Micronutrients: Adequate intake of iron, calcium, vitamin D, B vitamins is critical.
Duration:	Typically recommended for 2–4 weeks before surgery. Shorter durations (1–2 weeks) of a VLCD have been shown to significantly reduce liver size and improve operative safety. Although a longer duration (up to 4 weeks) may lead to greater weight loss and liver volume reduction. For patients with severe obesity (BMI >50 kg/m^2^) or metabolic complications, a longer preparation period of 4–6 weeks may be necessary to optimize conditions.
(C) Managing Comorbid Conditions [35,50,69,70,79]	Hypertension: Continue antihypertensive medication; individualize insulin and diuretic management
	OSA: Screen all patients, consider polysomnography for diagnosis.
	Gastrointestinal (GI) Pathologies: Preoperative EGD helps detect GI disorders and *H. pylori*.
	Mental health: Essential to address depression and anxiety.
Team Coordination	Multidisciplinary care involving surgeons, endocrinologists, nutritionists, psychologists, and other professionals optimizes outcomes.
(D) re-Surgical Exercise Recommendations [73,74,77]	Exercise modalities: Both moderate-intensity continuous training (MICT) and high-intensity interval training (HIIT) are effective. MICT reduces fat mass and increases muscle mass, while HIIT improves metabolic health by enhancing adiponectin levels. Program duration: Four weeks with 10 sessions (30 min each), performed 2–3 times per week. Benefits: Enhances aerobic capacity, insulin sensitivity, and lipid profiles, reducing surgical risks and aiding recovery.

EGD: Esophagogastroduodenoscopy; HIIT: High-intensity interval training; LCD: Low calorie diet; MICT: Moderate-intensity continuous training; OSA: Obstructive sleep apnea; VLCD: Very low-calorie diet.

**Table 2:** Gradual introduction of foods.

Length	Diet Type	Suggestions
0 to 3 days	Clear liquids [58,61]	Drink in small sips. No straw use. Liquids without gas, caffeine, lactose, fats, or alcohol.
From 7 to 14 days	Low-fat full liquid diet [58,61]	No bits and pieces. No sugar, caffeine, lactose, gas, fat, or alcohol. No straw. Protein supplement 25 to 30 g/meal. Vitamin and mineral supplement.
From day 7 to 14 and for 1 to 2 weeks according to evolution and tolerance.	High-protein, low-fat, and moderate carbohydrate diet (“purée”) [58,61]	Soft foods that do not require chewing. Low in fat and sugar. Modified fiber. Foods rich in protein. Fractionation, 3 to 5 feedings/day. Sufficient hydration. Suspend liquids 15 min before and 30 min after intake. Protein, vitamin, and mineral supplementation.
Approximately week 4 and for 1 to 2 weeks according to evolution and tolerance.	Hypocaloric-hyperproteic solid diet (soft foods) [58,61]	Cut into small pieces Correct chewing. Fiber from 10 to 14 g/day. At least 20 min for each ingestion.

**Table 3:** Comprehensive approach involving nutritional, psychological, and medical interventions to prevent weight regain after BS and improve long-term metabolic health.

Recommendation	Description
Personalized Interventions	Adopt personalized nutritional strategies focusing on high-quality foods such as fruits, vegetables, and lean proteins [132].
Regulated Energy Deficit	Maintain a controlled energy deficit to prevent weight gain, while avoiding nutritional deficiencies [132].
Balanced and High-Quality Diet	Promote balanced diets, such as the Mediterranean or DASH diets, that provide cardiometabolic benefits [132,157,158].
Micronutrient Supplementation	Supplement key nutrients (vitamins B12, D, and iron) to prevent deficiencies, especially in younger patients or those with mental health disorders [133,135,136,161]
Separation of Liquids and Solids	Maintain the separation between liquids and solids during meals to improve satiety and prevent overeating [134].
Prioritize Protein-Rich Foods	Favor protein-rich foods to improve satiety, preserve muscle mass, and control appetite [132,142].
Incorporate Fiber in the Diet	Increase fiber intake to improve metabolic health, control appetite, and reduce visceral fat [159].
Adherence to Continuous Medical Monitoring	Maintain regular follow-up consultations with medical and nutritional guidance to prevent metabolic complications and reinforce healthy habits [135,136].
Behavioral Interventions	Integrate behavioral strategies such as cognitive-behavioral therapy to improve adherence and address psychological factors contributing to weight gain [132,140].
Preoperative Education	Promote nutritional education before surgery to ensure sustainable eating habits and prevent long-term weight gain [132,135].
Monitoring Eating Habits	Continuously monitor and evaluate eating and psychological habits, such as emotional eating or grazing behaviors, to address factors contributing to weight regain [140].
